# Antibiotic resistance and molecular characterization of the hydrogen sulfide-negative phenotype among diverse *Salmonella* serovars in China

**DOI:** 10.1186/s12879-018-3209-3

**Published:** 2018-07-03

**Authors:** Jing Xie, Fuli Wu, Xuebin Xu, Xiaoxia Yang, Rongtao Zhao, Qiuxia Ma, Peng Li, Ligui Wang, Rongzhang Hao, Leiji Jia, Xinying Du, Shaofu Qiu, Hongbin Song

**Affiliations:** 1 Institute of Disease Control and Prevention, Academy of Military Medical Sciences, 20 Dongda Street, Fengtai District, Beijing, 100071 China; 2 Shanghai Municipal Center for Disease Control and Prevention, Shanghai, China

**Keywords:** Multidrug resistance, Hydrogen sulfide, *Salmonella*, Serogroup, *phs* operon

## Abstract

**Background:**

Among 2179 *Salmonella* isolates obtained during national surveillance for salmonellosis in China from 2005 to 2013, we identified 46 non-H_2_S-producing strains originating from different sources.

**Methods:**

The isolates were characterized in terms of antibiotic resistance and genetic variability by pulsed-field gel electrophoresis and multilocus sequence typing. Mutation in the *phs* operon, which may account for the non-H_2_S-producing phenotype of the isolated *Salmonella* strains, was performed in this study.

**Results:**

Among isolated non-H_2_S-producing *Salmonella* strains, more than 50% were recovered from diarrhea patients, of which H_2_S-negative *S.* Gallinarum, *S.* Typhimurium, *S.* Choleraesuis and *S.* Paratyphi A isolates constituted 76%. H_2_S-negative isolates exhibited a high rate of resistance to ticarcillin, ampicillin, and tetracycline, and eight of them had the multidrug resistance phenotype. Most H_2_S-negative *Salmonella* isolates had similar pulsed-field gel electrophoresis profiles and the same sequence type as H_2_S-positive strains, indicating a close origin, but carried mutations in the *phsA* gene, which may account for the non-H_2_S-producing phenotype.

**Conclusions:**

Our data indicate that multiple H_2_S-negative strains have emerged and persist in China, emphasizing the necessity to implement efficient surveillance measures for controlling dissemination of these atypical *Salmonella* strains.

**Electronic supplementary material:**

The online version of this article (10.1186/s12879-018-3209-3) contains supplementary material, which is available to authorized users.

## Background

*Salmonella* remains one of the most prevalent foodborne pathogens causing bacterial gastroenteritis [1, 2]. Infection through contaminated food and water can lead to diarrhea and even death. It has been reported that *Salmonella* species can account for nearly 93.8 million cases of gastroenteritis every year worldwide, resulting in 155,000 deaths [3]. In China, *Salmonella* spp. are responsible for approximately 22.2% of foodborne diseases, resulting in 9.03 million cases and estimated 800 deaths annually [4]. Therefore, it is particularly important to further strengthen the surveillance and control of *Salmonella*. As hydrogen sulfide (H_2_S) production is characteristic of these pathogens, H_2_S detection has become a screening method to identify and differentiate *Salmonella* from other intestinal bacteria [5]. However, H_2_S-negative *Salmonella* isolates have been continuously reported in different countries; thus, one isolate was identified in Kuwait, 10 in United States, 31 in Japan, and 58 in Southeast China [6–12]. In our previous studies, we also identified 43 H_2_S-negative *Salmonella* isolates during national surveillance of salmonellosis in China [13–15]. These results suggest that the occurrence of the atypical H_2_S-negative *Salmonella* variants is growing throughout the world.

Increasing resistance of *Salmonella* to antibiotics, especially high prevalence of multidrug resistance (MDR), is a global concern. In many regions of the world, a high resistance rate to conventional antimicrobial agents has been reported for H_2_S-positive *Salmonella* [16–19]*.* Although H_2_S-negative *Salmonella* isolates may be highly sensitive to a multitude antibiotics owning to H_2_S defending bacteria against oxidative stress imposed by antibiotics [20], there is increasing evidence that high resistance rate to antibiotics was also observed in many H_2_S-negative *Salmonella* isolates [6, 7, 14]. Importantly, extended-spectrum cephalosporins and fluoroquinolones have been widely used as alternative agents for treatment of salmonellosis. However, a non-H_2_S-producing *S.* Kentucky isolate had the MDR phenotype, including resistance to ciprofloxacin, whereas non-H_2_S-producing *S*. Infantis and *S*. Typhimurium expressed CMY-2β-lactamase and had reduced susceptibility to cefazolin [6, 8]. These results may due to different bactericidal mechanisms of various classes of antibiotics. Taken together, it is important to pay more attention on the surveillance of H_2_S-negative *Salmonella*.

*Salmonella* spp. produce H_2_S from various enzymes encoded by different operons, such as *phs* operon, *cysJIH* operon and *asr* operon [21–23]. However, the *phs* operon is essential for this activity in *Salmonella* [24]. Sakano et al. [8] detected a nonsense mutation in the *phsA* gene in H_2_S-negative *S.* Infantis and *S.* Typhimurium isolates, and in our previous studies, we found more mutations in the *phsA* gene of H_2_S-negative *S.* Senftenberg, *S.* Choleraesuis, and *S.* Aberdeen [13–15]. These data suggest that the disruption of the *phs* locus is responsible for the lack of H_2_S production and incorrect identification of *Salmonella*. Here, we report the identification of 46 H_2_S-negative *Salmonella* strains during the national surveillance of salmonellosis from 2005 to 2013 in China. Our findings indicate that various serovars of H_2_S-negative *Salmonella* have emerged in China. Therefore, effective measures should be urgently taken to prevent and control further dissemination of H_2_S-negative *Salmonella* in China.

## Methods

### Isolation, identification, and serotyping of *Salmonella* strains

In this study, *Salmonella* isolates were obtained during national surveillance for salmonellosis in China conducted from 2005 to 2013. Samples were collected in eight cities throughout China (Beijing, Nanjing, Shanghai, Guangzhou, Shenyang, Jinan, Xinjiang, and Yuxi) from various sources, including diarrhea patients, poultry, livestock, vegetables, aquatic products, and water. First, the samples were enriched by culturing in Selenite Brilliant Green broth (CHROMagar, Shanghai, China) at 37 °C for 16–22 h, and then plated on xylose lysine deoxycholate agar (XLD; CHROMagar) and CHROMagar Salmonella medium (CAS; CHROMagar) at 37 °C for 18–24 h. Colonies suspected to be formed by *Salmonella* were serotyped by slide agglutination tests (SSI Diagnostica, Hillerød, Denmark). API 20E test strips (bioMerieux Vitek, Marcy-l’Etoile, France) were used to confirm the identified colonies and examine for the H_2_S-producing phenotype.

### Antimicrobial susceptibility testing

H_2_S-negative and H_2_S-positive *Salmonella* isolates were tested for sensitivity to 21 antibiotics used commonly in laboratories and hospitals. MICs of 21 antibiotics including amikacin, ampicillin, aztreonam, cefazolin, cefepime, cefoperazone, cefoxitin, ceftazidime, ceftriaxone, chloramphenicol, gentamicin, imipenem, levofloxacin, nitrofurantoin, norfloxacin, piperacillin, tetracycline, ticarcillin, ticarcillin-clavulanic acid, tobramycin, and trimethoprim-sulfamethoxazole were evaluated by the automated broth microdilution method using 96-well microtiter plates (Sensititre; Terk Diagnostic Systems, Thermo Fisher Scientific Inc., Cleveland, OH, USA). The results were interpreted according to the recommendations of the Clinical and Laboratory Standards Institute (CLSI) [25] and *Escherichia coli* strain ATCC 25922 was used for quality control. Genetic variations related to molecular mechanisms responsible for the resistance to quinolones were examined by PCR. The specific primers were as follows: *gyrA* forward 5′-TTATGCGATGTCGGTCATTGTT-3′ and reverse 5′-TTCACCAGCTCGGCGATT-3′ and *parC* forward 5′-CGTGCGTTGCCGTTTATTG-3′ and reverse 5′-CAACTGATCCAGCGTCGTT-3′.

### Multilocus sequence typing analysis

Total DNA was extracted from the identified *Salmonella* isolates using the TIANamp Bacteria DNA kit (Tiangen Biotech, Beijing, China) according to the manufacturer’s instruction, and MLST was performed using the protocol described in our previous study [13]. Sequences of seven housekeeping genes (*aroC*, *dnaN*, *hemD*, *hisD*, *purE*, *sucA*, and *thrA*) were downloaded from the MLST database. The sequences of PCR-amplified products were uploaded to http://enterobase.warwick.ac.uk/species/senterica/allele_st_search for comparison and analysis to determine the sequence type (ST).

### Pulsed-field gel electrophoresis analysis

DNA was digested with *Xba*I (Takara, Dalian, Japan) at 37 °C for 3 h and subjected to PFGE according to a standardized protocol [26]. Then, electrophoresis of the digested DNA was carried out using a CHEF Mapper PFGE system (Bio-Rad, Hercules, CA, USA) in 1% SeaKem agarose and 0.5× Tris-borate-EDTA for 19 h with the following run parameters: 6 V/cm and a linear increase in switching times from 2.16 to 63.8 s. Macrorestriction patterns were compared and analyzed using the BioNumerics Fingerprinting software version 6.0 (Applied-Maths, Sint-Martens-Latem, Belgium). Dendrograms were constructed according to the unweighted pair-group method of arithmetic average (UPGMA), and the Disc coefficient of similarity was determined based on 1.2% position tolerance. *S.* Braenderup H9812 was used as a standard [27].

### Amplification and sequencing of the *Phs* operon

The *phs* operon containing three open reading frames, designated *phsA*, *phsB*, and *phsC*, which encode thiosulfate reductases catalyze thiosulfate to H_2_S. The *phs* operon (*phsA*, *phsB*, and *phsC*) was amplified by PCR and sequenced by Sangon Biotech. The specific primers were as follows: *phsA1* forward 5′-CGTTGGATGCCTGTTCAG-3′ and reverse 5′-AGGTCGTAGAGCCGATTG-3′, *phsA2* forward5′-CGCCGTTCAACTGATAGA-3′ and reverse 5′-AATGGTGAGCTTCGATCC-3′, *phsA3* forward 5′-CATCGTAGAGCTGTTCATCA-3′ and reverse 5′-CATGTGCGTGTTCAGGAA-3′, *phsB* forward 5′-CAAGCATGAGCAGCACCAC-3′ and reverse 5′-ATGAGGGAGGAGGGAACCAT-3′, and *phsC* forward 5′-GATGGTCTCTATTTGCCGTTCT-3′ and reverse 5′-GGTGCTGCTCATGCTTGTT-3′.

The PCR amplification conditions were as follows: PCR conditions were as follows: 95 °C for 5 min; 30 cycles of 95 °C for 30 s, 57 °C for 40 s, and 72 °C for 45 s; and 72 °C for 7 min, using Ex Taq DNA polymerase (TaKaRa/Clontech). The results were imported into DNAman 6.0, and genetic differences were detected using MEGA version 7.0. Reference strains for *phs* operon sequence analysis are listed in Additional file 1: Table S1. For *S.* Derby and *S.* Meleagridis, the reference strains (*S.* Derby str. 91,780 and *S.* Meleagridis str. SH10SF424–1) were of the H_2_S-positive phenotype identified in this study.

### Nucleotide sequence accession numbers

The nucleotide sequences obtained in this study have been deposited to the NCBI database; GenBank accession numbers are listed in Additional file 1: Table S2.

### Statistical analysis

The data were analyzed by chi-square test using the SPSS software (SPSS Inc., Chicago, IL, USA; version 17.0), and a *P*-value < 0.05 was considered to indicate statistically significant differences.

## Results

### H_2_S-negative *Salmonella* isolates

**Fig. 1:**
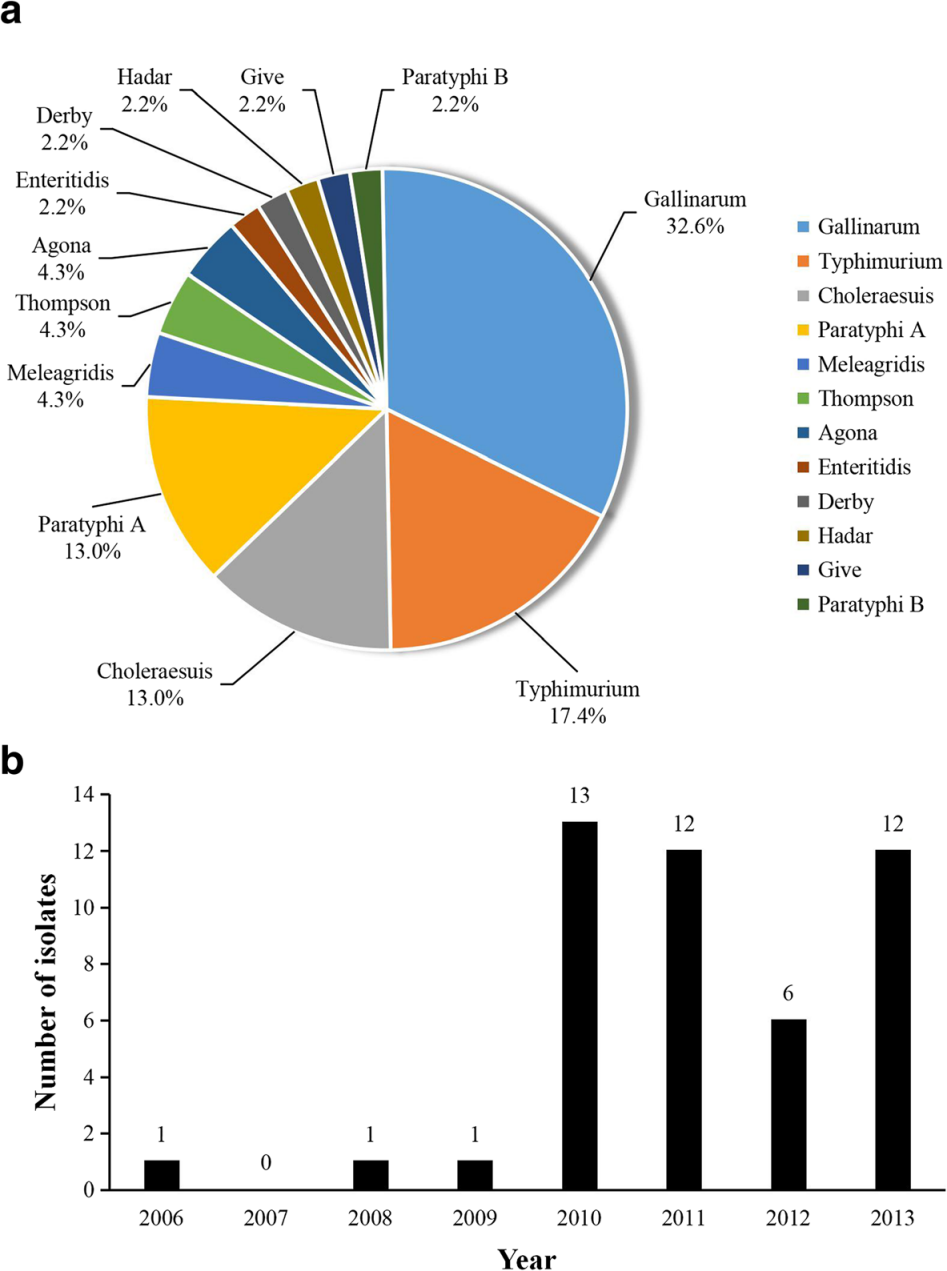
Distribution of non-H_2_S-producing *Salmonella* isolates in China by serotype (**a**) and time of isolation (**b**)

During national surveillance of salmonellosis in 2005–2013 in China, 46 H_2_S-negative *Salmonella* isolates were identified among 2179 *Salmonella* strains from various sources. These strains were divided into 12 serovars: *S.* Gallinarum, *S.* Typhimurium, *S.* Choleraesuis, *S.* Paratyphi A, *S.* Meleagridis, *S.* Agona, *S.* Thompson, *S.* Enteritidis, *S.* Derby, *S.* Paratyphi B, *S.* Hadar, and *S.* Give (Fig. 1a). Among them, *S.* Gallinarum, *S.* Paratyphi A, *S.* Meleagridis, *S.* Agona, *S.* Thompson, *S.* Paratyphi B, *S.* Hadar, and *S.* Give were reported as having the non-H_2_S-producing phenotype for the first time. The most prevalent serovars for non-H_2_S-producing *Salmonella* were *S.* Gallinarum, *S.* Typhimurium, *S.* Choleraesuis and *S.* Paratyphi A, accounting for 33, 17, 13 and 13%, respectively, of the entire collection. Twenty-four (52%) samples were from humans with diarrhea, whereas 21 (46%) were from animals, including pork, chicken, and aquatic products, and one H_2_S-negative isolate was recovered from the river. During the period from 2006 to 2009, only three H_2_S-negative isolates were identified; however, approximately 93% isolates were identified with a high level of detection in the following 4 years (Fig. 1b).

### Antimicrobial resistance among H_2_S-negative *Salmonella*

Testing of the identified *Salmonella* isolates for antimicrobial susceptibility to 21 different antibiotics showed that they exhibited a high rate of resistance to conventional antimicrobial agents. H_2_S-negative isolates displayed high resistance to ampicillin, ticarcillin, tetracycline, tobramycin, chloramphenicol, gentamicin, and trimethoprim-sulfamethoxazole (Table 1); furthermore, quinolone-resistant and cefazolin-resistant strains were also detected.

**Table 1:** Antibiotic resistance patterns for various serovars of H_2_S-negative *Salmonella* isolates

Antimicrobial drugs	Resistant isolates, % (n)						
	Total (*n* = 46)	Gallinarum (*n* = 15)	Typhimurium (*n* = 8)	Choleraesuis (*n* = 6)	Meleagridis (*n* = 2)	Derby (*n* = 1)	Give (*n* = 1)
Cefazidime	0	0	0	0	0	0	0
Ceftriaxone	0	0	0	0	0	0	0
Cefepime	0	0	0	0	0	0	0
Cefoperazone	0	0	0	0	0	0	0
Imipenem	0	0	0	0	0	0	0
Nitrofurantoin	2 (1)	0	13 (1)	0	0	0	0
Piperacillin	4 (2)	0	13 (1)	17 (1)	0	0	0
Ticarcillin	46 (21)	80 (12)	50 (4)	67 (4)	0	0	100 (1)
Ticarcillin-clavulanic acid	7 (3)	6 (1)	25 (2)	0	0	0	0
Ampicillin	46 (21)	80 (12)	50 (4)	67 (4)	0	0	100 (1)
Tetracycline	35 (16)	13 (2)	63 (5)	83 (5)	100 (2)	100 (1)	100 (1)
Cefazolin	2 (1)	7 (1)	0	0	0	0	0
Cefoxitin		0	0	0	0	0	0
Aztreonam		0	0	0	0	0	0
Chloramphenicol	15 (7)	0	25 (2)	50 (3)	0	100 (1)	100 (1)
Tobramycin	2 (1)	0	0	0	0	0	100 (1)
Gentamicin	13 (6)	0	25 (2)	50 (3)	0	0	100 (1)
Amikacin	0	0	0	0	0	0	0
Trimethoprim-sulfamethoxazole	24 (11)	6 (1)	25 (2)	67 (4)	100 (2)	100 (1)	100 (1)
Norfloxacin	4 (2)	0	25 (2)	0	0	0	0
Levofloxacin	4 (2)	0	25 (2)	0	0	0	0

Each serovar showed a distinct antibiotic resistance pattern (Table 1). Overall, H_2_S-negative *S.* Typhimurium isolates demonstrated resistance to 11 antibiotics, with a high resistance rate to penicillins and tetracyclines. For two quinolones-resistant *S.* Typhimurium isolates, multisite mutations were detected simultaneously in the *gyrA* and *parC* genes; polymorphisms at positions 200 and 250 may be responsible for the resistance to quinolones (GenBank accession numbers: KY814731–KY814732 and KY814737–KY814738). H_2_S-negative *S.* Choleraesuis isolates displayed high resistance to tetracycline, ticarcillin, ampicillin, trimethoprim-sulfamethoxazole, chloramphenicol and gentamicin; importantly, all these isolates were from hospitalized diarrhea patients. About 80% of H_2_S-negative *S.* Gallinarum isolates were resistant to ticarcillin and ampicillin. In addition, H_2_S-negative *S.* Give, *S.* Derby, and *S.* Meleagridis were resistant to seven, three, and two antibiotics, respectively. All H_2_S-negative *S.* Paratyphi A, *S.* Paratyphi B, *S.* Enteritidis, *S.* Agona, *S.* Harder, and *S.* Thompson isolates were susceptible to the 21 tested antimicrobials.

Among the examined H_2_S-negative *Salmonella*, the MDR phenotype was observed in eight (17%) isolates (Table 2). The H_2_S-negative MDR strains showed seven distinct antibiotic-resistance profiles. Two MDR isolates identified as H_2_S-negative *S.* Typhimurium had the widest antibiotic-resistance profiles, showing resistance to more than six classes of antimicrobials, including 11 individual antibiotics. H_2_S-negative *S.* Choleraesuis isolates displayed the highest MDR rate: 67% (4 of 6). Among them, one isolate was resistant to five classes of antimicrobials, and the others were resistant to four classes. Moreover, H_2_S-negative *S.* Give and *S.* Derby isolates also had the MDR phenotype. The same proportions of H_2_S-negative MDR isolates (about 50%) were recovered from humans and animals.

**Table 2:** Multidrug resistance profiles of eight H_2_S-negative MDR isolates

Serotype	Number of isolates	Antibiotic resistance profiles	Antimicrobial drug classes						
			Aminoglycosides	Amphenicols	Folate pathway inhibitors	Nitrofurans	Penicillins	Quinolones	Tetracyclines
Typhimurium	1	GEN/CHL/SXT/NIT/AMP/TIC/TIM/LEV/NOR/TET	+	+	+	+	+	+	+
Typhimurium	1	GEN/CHL/SXT/AMP/PIP/TIC/TIM/LEV/NOR/TET	+	+	+		+	+	+
Give	1	GEN/TOB/CHL/SXT/AMP/TIC/TET	+	+	+		+		+
Choleraesuis	1	GEN/CHL/SXT/AMP/PIP/TIC/TET	+	+	+		+		+
Choleraesuis	2	GEN/SXT/AMP/TIC/TET	+		+		+		+
Choleraesuis	1	CHL/SXT/AMP/TIC/TET		+	+		+		+
Derby	1	CHL/SXT/TET		+	+				+

*AMP* ampicillin, *CHL* chloramphenicol, *GEN* gentamicin, *LEV* levofloxacin, *NIT* nitrofurantoin, *NOR* norfloxacin, *PIP* piperacillin, *SXT* trimethoprim/sulfamethoxazole, *TET* tetracycline, *TIC* ticarcillin, *TIM* ticarcillin/clavulanic acid, *TOB* tobramycin

### PFGE and MLST analyses

**Fig. 2:**
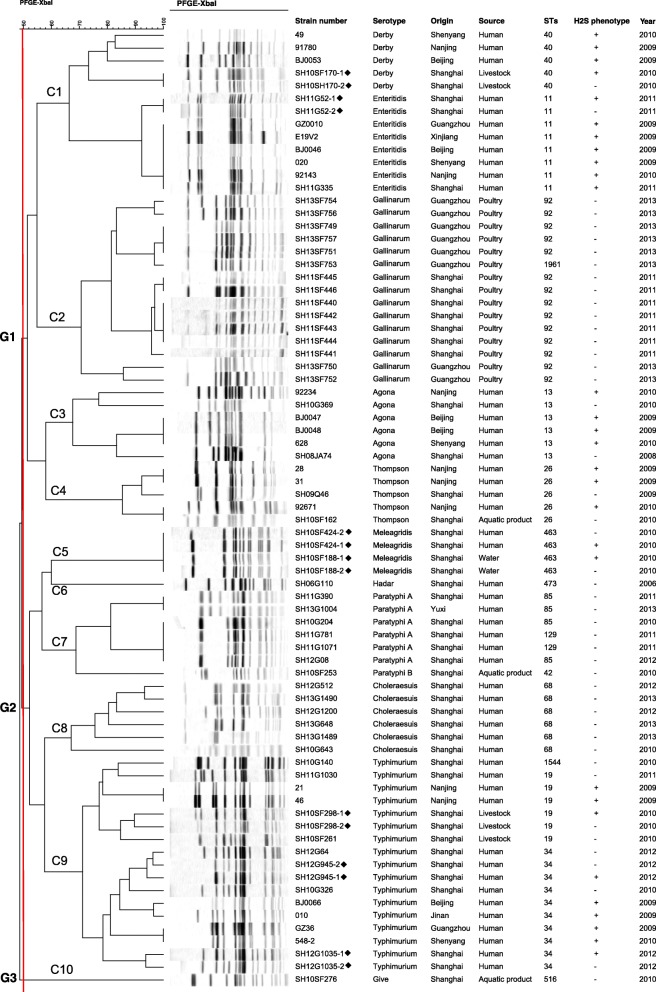
Dendrogram analysis based on the PFGE data for 75 *Salmonella* isolates. Strain number, serotype, origin, source, ST, and year of isolation are listed after each PFGE profile. Diamonds indicate strains isolated from the same sample

H_2_S-positive *S.* Gallinarum, *S.* Hadar, *S.* Paratyphi A, *S.* Paratyphi B, *S.* Choleraesuis, and *S.* Give isolates were not detected during national surveillance for salmonellosis in this study. For PFGE testing and MLST analysis, we combined 46 H_2_S-negative and 29 H_2_S-positive *Salmonella* isolates (four *S.* Derby, seven *S.* Enteritidis, four *S.* Agona, three *S.* Thompson, two *S.* Meleagridis, and nine *S.* Typhimurium) to clarify their genetic relationships. Cluster analysis divided the 75 isolates into three distinct groups sharing approximately 50% similarity (Fig. 2).

Group I consisted of four clusters. Cluster 1 contained two serovars: *S.* Derby and *S.* Enteritidis. *S.* Derby H_2_S-producing SH10SF170–1 and non-H_2_S-producing SH10SF170–2 had indistinguishable PFGE profiles, similar to *S.* Enteritidis SH11G52–1 and SH11G52–2. In cluster 2, all isolates were from poultry and were identified as H_2_S-negative *S.* Gallinarum. Although these strains were acquired from different places at different times, they shared high similarity in their PFGE patterns. All H_2_S-negative *S.* Gallinarum isolates belonged to ST92 except for one belonging to the new ST1961, which was a single locus variant (SLV) of ST92. Moreover, *S.* Agona H_2_S-negative isolates had PFGE profiles similar to those of H_2_S-positive strains, as well as to that of H_2_S-negative *S.* Thompson. *S.* Derby, *S.* Enteritidis, *S.* Agona, and *S.* Thompson belonged to ST40, ST11, ST13, and ST26, respectively.

In Group II, pairs of H_2_S-producing and -non-producing *S.* Meleagridis isolated from two samples were grouped in cluster 5; despite different sources, the two pairs had the same PFGE patterns and ST. Cluster 6 contained only one H_2_S-negative *S.* Hadar belonging to ST473. Cluster 7 was formed by six H_2_S-negative *S.* Paratyphi A and one H_2_S-negative *S.* Paratyphi B isolates. The six H_2_S-negative *S.* Paratyphi A isolates (four ST85 and two ST129) were from humans, andoneH_2_S-negative *S.* Paratyphi B belonging to ST42 was from an aquatic product. The six H_2_S-negative *S.* Choleraesuis isolates in cluster 8 had close PFGE patterns, the same ST, and were acquired from the same source and place. In cluster 9, pairs SH10SF298–1 and SH10SF298–2, SH12G945–1 and SH12G945–2, and SH12G1035–1 and SH12G1035–1 were from the same samples, respectively; each pair had very similar banding patterns. Among *S.* Typhimurium H_2_S-negative isolates, six were from humans: four ST34, one ST1544, and one ST19; the former two were SLVs of ST19 and double locus variants (DLVs) of each other.

Finally, Group III consisted of one H_2_S-negative *S.* Give isolate belonging to ST516.

### Sequence analysis of the *Phs* operon

Previous studies have reported mutations in the *phs* operon may responsible for the non-H_2_S-producing phenotype in *Salmonella* [8, 13–15]*.* In the *phsA* gene, three different mutation sites were detected among the 46 H_2_S-negative *Salmonella* isolates (Table 3). In *S.* Gallinarum isolates, missense mutation 1624C > T leading to the Leu > Phe substitution was found. In *S.* Choleraesuis isolates, single base deletion 760delG was detected, leading to a frameshift mutation. One H_2_S-negative *S.* Typhimurium (SH10G140) isolate had mutation 1087delA, which caused a frameshift and change in the amino acid sequence. Only few missense mutations were identified in H_2_S-negative *S.* Give and *S.* Hadar (data not shown). However, no mutations in the *phsA* gene were detected in the remaining 22 isolates (seven *S.* Typhimurium, six *S.* Paratyphi A, two *S.* Agona, two *S.* Meleagridis, two *S.* Thompson, one *S.* Paratyphi B, one *S.* Derby, and one *S.* Enteritidis).

**Table 3:** Mutations detected in the *phsA* gene of H_2_S-negative *Salmonella* isolates

Serotype	Number of isolates	Mutation	Mutation type
Gallinarum	15	**1624C > T**	Missense
Choleraesuis	6	760delG	Frameshift
Typhimurium	1	**1087delA**	Frameshift

New mutations are marked bold

In the *phsB* gene, we identified only one nonsense mutation in a *S.* Hadar isolate and multiple missense mutations in *S*. Gallinarum isolates. There were four main missense mutation sites, including substitutions 164 T > C (eight isolates), 314G > C (eight isolates), 319C > A (11 isolates), and 373C > T (eight isolates). In the *phsC* gene, nonsense and missense mutations were found in one H_2_S-negative *S.* Typhimurium and three H_2_S-negative *S.* Paratyphi A isolates, respectively (Additional file 1: Table S3).

Sixteen H_2_S-negative isolates carried no mutations in the *phs* locus (*phsA*, *phsB*, and *phsC* genes).

## Discussion

Although there are few reports about H_2_S-negative *Salmonella*, the incidence of H_2_S-negative strains is on the rise lately. To the best of our knowledge, 100 H_2_S-negative *Salmonella* isolates of 13 serovars have been reported [6–12]. Moreover, 17 H_2_S-negative *S.* Senftenberg isolates, 19 H_2_S-negative *S.* Choleraesuis isolates, and seven H_2_S-negative *S.* Aberdeen isolates were reported in our previous studies [13–15]. In this study, a total of 46 H_2_S-negative *Salmonella* strains belonging to 12 various serovars were isolated from diverse sources across China during 2005–2013. H_2_S-negative *S.* Agona, *S.* Meleagridis, *S.* Gallinarum, *S.* Give, *S.* Hadar, *S.* Paratyphi A, *S.* Paratyphi B, and *S.* Thompson were newly identified, indicating that multiple *Salmonella* serovars could present the non-H_2_S-producing phenotype. Notably, our surveillance data revealed that 52% strains were isolated from diarrhea patients in hospitals, suggesting that H_2_S-negative *Salmonella* isolates, similar to H_2_S-positive strains, may play an important role in causing human infections. In addition, food products were another important source of H_2_S-negative *Salmonella* isolates. It has been reported that 33 *Salmonella* isolates were identified as H_2_S-negative in 82 retail meat samples from markets in Shenzhen, China [10]. In this study, about 46% H_2_S-negative *Salmonella* isolates were from pork, chicken, and aquatic products, suggesting that H_2_S-negative *Salmonella* could be present in various foods. Since *Salmonella* isolation methods vary among laboratories and hospitals from different locations, it is possible that the number of H_2_S-negative isolates could be higher than that reported here. Therefore, proposing a standard screening procedure will reduce the missing H_2_S-negative *Salmonella* during laboratory and hospital screening*.* In addition, we recommend using API 20E biochemical test kits and serological testing to further confirm the suspected H_2_S-negative colonies when necessary.

Although a large number of H_2_S-negative *Salmonella* strains have been reported, their antibiotic resistance patterns were not clarified. Among the H_2_S-negative *Salmonella* identified in this study, *S.* Choleraesuis exhibited a high rate of antibiotic resistance, comprising 67% of MDR isolates; similar data on the MDR rate among H_2_S-negative *S.* Choleraesuis isolates were reported in Japan and in our previous study [7, 14]. To the best of our knowledge, MDR has been previously detected only in H_2_S-negative *S.* Choleraesuis and *S.* Kentucky [6, 7]. In this study, the MDR phenotype was observed among H_2_S-negative *S.* Typhimurium, *S.* Give, and *S.* Derby isolates, which were resistant to 11, seven, and three antibiotics, respectively. In addition, amino acid changes in the GyrA and ParC proteins have been detected in the ciprofloxacin-resistant H_2_S-negative *S.* Kentucky strain and norfloxacin-resistant H_2_S-negative*S.* Choleraesuis strains [6]. In this study, we detected mutations in the *gyrA* and *parC* genes of H_2_S-negative *S.* Typhimurium strains with complete resistance to quinolones. Cumulatively, these results suggest that the emergence of antibiotic resistance among H_2_S-negative *Salmonella* strains presents a more serious problem than has been previously anticipated. Although the mechanism of H_2_S-mediated antibiotic resistance has been demonstrate in several bacteria, this process requires anaerobic conditions and antibiotics which have to exert their bactericidal effect by oxidative stress [20, 23, 28]. What’s more, plasmid-mediated drug resistance mechanism is responsible for the increased resistance rate to antibiotics as well. Hence, there is a great need to take effective measures to control the prevalence of H_2_S-negative *Salmonella* isolates with MDR.

Disruption of the *phsA* gene seems to underlie the lack of H_2_S production in a large number of H_2_S-negative *Salmonella* isolates, although mutation analysis was not conducted for all reported H_2_S-negative *Salmonella* [8, 13–15]. H_2_S-negative *S.* Typhimurium and *S.* Infantis have been reported to contain nonsense mutations at positions 1440 and 358 of the *phsA* gene, respectively [8]. Previously, we identified a frameshift mutation in H_2_S-negative *S.* Choleraesuis and nonsense mutations in H_2_S-negative *S.* Aberdeen and *S.* Senftenberg [13–15]. In this study, 22 (48%) H_2_S-negative *Salmonella* isolates carried mutations at different positions of the *phsA* gene, indicating that this gene may be responsible for the atypical H_2_S phenotype. Moreover, we found that *phsA* mutation sites were serovar-specific, suggesting that serovars containing H_2_S-negative isolates have distinct genetic mechanisms leading to mutations in the *phs* locus. As the *phs* operon is essential for the production of H_2_S from thiosulfate under anaerobic conditions, thiosulfate would concentrate around H_2_S-negative *Salmonella* cells and react with oxygen species generated during inflammation, producing a new respiratory electron acceptor tetrathionate [29–32]. These studies suggest that the accumulation of thiosulfate by H_2_S-negative *Salmonella* strains, including *S.* Typhimurium, may provide these strains a growth advantage in competition with other bacteria in the gut lumen, thus presenting a reasonable explanation for the large number of H_2_S-negative *Salmonella* isolates with high rate of antibiotic resistance detected in humans.

## Conclusion

We identified 46 H_2_S-negative *Salmonella* isolates belonging to 12 serovars in China. As the number of these *Salmonella* strains has been rapidly increasing over a short period of 9 years, the emergence and prevalence of H_2_S-negative *Salmonella* cannot be ignored, and special attention should be paid to avoid their further dissemination by implementing specific surveillance measures.

## Additional file

Additional file 1: **Table S1.** Reference strains for *phs* operon sequence analysis. **Table S2.** GenBank accession numbers for *phs* operon sequences from 46 H_2_S-negative *Salmonella* isolates. **Table S3.** Mutations detected in the *phsB* and *phsC* genes of H_2_S-negative *Salmonella* isolates. (DOC 71 kb)

## Abbreviations

MDR: Multidrug resistance
MLST: Multilocus sequence typing
PFGE: Pulsed-field gel electrophoresis
