# Supplementary material for: Antibiotic resistance and molecular characterization of the hydrogen sulfide-negative phenotype among diverse Salmonella serovars in China
Source: BMC Infect Dis. 2018 Jul 3;18:292. doi: 10.1186/s12879-018-3209-3 (PMC6029346; doi:10.1186/s12879-018-3209-3)
Supplement: Supplementary file 1 — Table S1. Reference strains for phs operon sequence analysis. Table S2. GenBank accession numbers for phs operon sequences from 46 H2S-negative Salmonella isolates. Table S3. Mutations detected in the phsB and phsC genes of H2S-negative Salmonella isolates. (DOC 71 kb) [file 12879_2018_3209_MOESM1_ESM.doc]

**Table S1. Reference strains for *phs*** operon sequence analysis

| **Serotype** | **Reference strain** | **GenBank accession number** |
| --- | --- | --- |
| Agona | *S*. Agona str. SL483 | NC_011149.1 |
| Choleraesuis | S.Choleraesuis str. SC-B67 | NC_006905.1 |
| Derby | S. Derby str. 91780 | KY211924 |
| Enteritidis | S. Enteritidis str. SA20094177 | CP007468.2 |
| Gallinarum | S. Gallinarum str. 287/91 | NC_011274.1 |
| Give | S.Bredeney str. CFSAN001080 | CP007533.1 |
| Hadar | S. Saintpaul str. SARA26 | CP017727.1 |
| Meleagridis | S.Meleagridis str. SH10SF424-1 | KY211925 |
| Paratyphi A | S. Paratyphi A str. ATCC 9150 | NC_006511.1 |
| Paratyphi B | S. Paratyphi B str. SPB7 | NC_010102.1 |
| Thompson | S. Thompson strain RM1984 | CP012513.1 |
| Typhimurium | S.Typhimurium str. LT2 | NC_003197.1 |

**Table S2. GenBank accession numbers for *phs* operon sequences from 46 H_2_S-negative *Salmonella* isolates**

| **Strain number** | **GenBank accession number** |
| --- | --- |
| SH11G52-2 | KY211927 |
| SH13SF749 | KY211928 |
| SH13SF750 | KY211929 |
| SH13SF751 | KY211930 |
| SH13SF752 | KY211931 |
| SH13SF753 | KY211932 |
| SH13SF754 | KY211933 |
| SH13SF756 | KY211934 |
| SH13SF757 | KY211935 |
| SH10G643 | KY211936 |
| SH12G512 | KY211937 |
| SH12G648 | KY211938 |
| SH12G1200 | KY211939 |
| SH13G1489 | KY211940 |
| SH13G1490 | KY211941 |
| SH10 140 | KY211942 |
| SH10SF261 | KY211943 |
| SH10G326 | KY211944 |
| SH10SF298-2 | KY211945 |
| SH11G1030 | KY211946 |
| SH12G64 | KY211947 |
| SH12G945-2 | KY211948 |
| SH12G1035-2 | KY211949 |
| SH11G390 | KY211950 |
| SH13G1004 | KY211951 |
| SH10G204 | KY211952 |
| SH11G781 | KY211953 |
| SH11G1071 | KY211954 |
| SH12G08 | KY211955 |
| SH10SF253 | KY211956 |
| SH11SF440 | KY211957 |
| SH11SF441 | KY211958 |
| SH11SF442 | KY211959 |
| SH11SF443 | KY211960 |
| SH11SF444 | KY211961 |
| SH11SF445 | KY211962 |
| SH11SF446 | KY211963 |
| SH10SF424-2 | KY211965 |
| SH10SF188-2 | KY211966 |
| SH10SF170-2 | KY211967 |
| SH08JA74 | KY211968 |
| SH10SF369 | KY211969 |
| SH10SF162 | KY211970 |
| SH09Q46 | KY211971 |
| SH06G110 | KY211972 |
| SH10SF276 | KY211973 |

**Table S3. Mutations detected in the *phsB* and *phsC* genes of H_2_S-negative *Salmonella* isolates**

| **Gene** | **Serotype** | **Mutation** | **Mutation type** | **Number of isolates** |
| --- | --- | --- | --- | --- |
| *phsB* | Gallinarum | 319C>A | Missense | 11 |
|  |  | 164T>C | Missense | 8 |
|  |  | 314G>C | Missense | 8 |
|  |  | 373C>T | Missense | 8 |
|  | Hadar | 61C>A | Nonsense | 1 |
| *phsC* | Typhimurium | 577C>T | Nonsense | 1 |
|  | Paratyphi A | 745A>G | Missense | 3 |
